# Planning strategies for inter-fractional robustness in pancreatic patients treated with scanned carbon therapy

**DOI:** 10.1186/s13014-017-0832-x

**Published:** 2017-06-08

**Authors:** Vania Batista, Daniel Richter, Stephanie E. Combs, Oliver Jäkel

**Affiliations:** 1 Hospital for Radiooncology and Radiation Therapy, Heidelberg University Hospital, Heidelberg, Germany; 2 University Clinic of Erlangen, Erlangen, Germany; 3 Heidelberg Ion-Beam Therapy Center, Heidelberg, Germany; 4 Klinikum rechts der Isa, Technische Universität München, Muniche, Germany; 5 GSI Helmholtz Centre for Heavy Ion Research, Darmstadt, Germany; 6 Division Medical Physics in Radiation Oncology, German Cancer Research Center, Heidelberg, Germany

## Abstract

**Background:**

Managing inter-fractional anatomy changes is a challenging task in radiotherapy of pancreatic tumors, especially in scanned carbon-ion delivery. This treatment planning study aims to focus on clinically feasible solutions, such as the beam angle selection and margin design to increase the robustness against inter-fractional uncertainties.

**Methods:**

This study included 10 patients with weekly 3D-CT imaging and physician-approved Clinical Target Volume (CTV). The study was directed to keep the CTV-coverage using six beam angle configurations in combination with different Internal Target Volume (ITV) concepts. These were: geometric-margin (symmetric 3 and 5 mm margin); range-equivalent margins with an isotropic HU replacement; and to evaluate the need of asymmetric margins the water-equivalent range path (WEPL) was determined per patient from the set of CTs.

Plan optimization and forward dose calculation in each week-CT were performed with the research treatment planning system TRiP98 and the plan quality evaluated in terms of CTV coverage (V95_CTV_) and homogeneity dose (H_CTV_ = D5-D95).

**Results:**

The beam geometry had a substantial impact on the target irradiation over the treatment course, with the single posterior or two beams showing the best average coverage of the CTV. The use of geometric margins for the more robust beam geometries showed acceptable results, with a V95_CTV_ of (99.2 ± 1.2)% for the 5 mm-margin. For the non-robust configurations, due to substantial changes in the radiological depth, the use of this margin results in a V95_CTV_ that might be below 80%, only showing improvement when the range changes are included.

**Conclusions:**

Selection of adequate beam configurations and treatment margins in ion-beam therapy of pancreatic tumors is of great importance. For a single posterior beam or two beam configurations, application of geometrical margins compensate for dose degradation induced by inter-fractional anatomy changes for the majority of the analyzed treatment fractions.

**Electronic supplementary material:**

The online version of this article (doi:10.1186/s13014-017-0832-x) contains supplementary material, which is available to authorized users.

## Introduction

Pancreatic cancer is still a disease without an effective treatment option usually with low survival rates and local control [1]. For locally advanced tumors one of the treatment schemes is photon radio-chemotherapy. However, a recent analysis showed that most tumor recurrences occur within a 2 cm radius of the primary tumor [2], indicating the need of dose escalation to improve the local control while keeping reduced side-effects. Hence, an alternative is the use of ion-beam therapy with proton or heavy ions [3]. Studies from the National Institute for Radiological Sciences (NIRS) and at the Heidelberg Ion-Beam Therapy Center (HIT) investigate this assumption. NIRS studies about the dose escalation and combined-chemotherapy with carbon-ions radiotherapy have shown a strong increase in the tumor local control and in the overall survival [4]. These results have motivated the conduct of the Phoenix-Trial at HIT which intends to use scanned carbon-radiotherapy to downsize the tumor before surgery [5].

The physical characteristics of the carbon-ions beam offer the possibility for highly conformal treatments as consequence from the reduced lateral scattering, the finite range and the shape of the depth dose profile. Moreover, from a radiobiological point of view, carbon-ions exhibit a relative biological effectiveness (RBE) of 1.16-2.46 [6] for pancreatic tumor cells and a low oxygen enhancement ratio, which due to the large fraction of hypoxic cells in pancreatic tumors make them a promising treatment option [7].

However the finite range of ions is also a source of uncertainties. The high sensitivity of the ion range to density changes in the beam-path induces dose under- and overshoots. These variations can either result from inter-fractional anatomy changes, patient positioning or intra-fractional motion [3].

Inter-fractional changes in pancreatic patients are mainly due to tumor shrinkage organ filling (bowel and stomach) and loss of adipose tissue [8]. These variations can be included in the planning process through the use of safety margins, although this compromises the dose to the normal tissues and does not consider range changes. The selection of the beam angles also affects plan quality and should avoid density variations along the beam path, minimizing the variations of the water-equivalent-path-length (WEPL). Note, that the effect of tumour shrinkage is not avoided by these methods, but its influence in the neighboring OARs can be reduced through the use of robust beam configurations, and its monitoring will help in the identification of the need for plan adaptation.

Intra-fractional motion is caused by respiration [9] and bowel movement [10] which besides range changes might results in under- and over-dosage regions, as result from the interference between the beam delivery and the target motion (interplay) in scanned beam delivery systems.

This study focuses on the impact of inter-fractional motion on the delivered dose during the pancreatic treatment and on the development of clinically feasible strategies to increase the treatment robustness by optimizing beam angles and internal margins. The investigation of the effects of intra-fraction motion it is not within the scope of this work and will be the subject of future publications.

## Methods

### Patient dataset & imaging

For a set of ten patients which had already been treated with photon radiotherapy, an in-silico analysis of the impact of inter-fractional motion on the plan quality for the treatment with scanned carbon-ions was performed. All the patients were weekly CT imaged for positioning verification purposes.

The four CT images were registered to the first week-CT (considered here as the CT_plan_) through rigid registration. Registration was validated using anatomical landmarks and visual inspection.

### Contouring and volume definition

All weekly-CTs included physician-approved contours of the Gross Tumor Volume (GTV) and Clinical Target Volume (CTV). The CTV was defined as the GTV plus an isotropic margin of 5 mm. Details on the initial CTV volume of each patient and its variation in volume and location over the treatment are presented in the Additional file 1: Table S.1. To incorporate inter-fractional motion effects we investigated three approaches to define an Internal Target Volume (ITV) for dose optimization:

- **Geometrical ITV**_**G**_: Geometric concept by application of 3 and 5 mm symmetric margins to the CTV volumes, ITVG_3_ and ITVG_5_, respectively. Results were compared with the use of no additional margin (ITV_G0_).
- **Uniform range-margin ITV**_**HU**_: Based on the geometric concept (ITV_G_) a uniform range-margin was introduced into the optimization by replacing the HU values in an isotropic CTV-ITV margin of 3 or 5 mm with the patient-specific median density of the pancreas. The volumes were defined as ITV_HU3_ and ITV_HU5_. In addition, also the areas inside the ITV_HU_, in which the density was less than twice the standard deviation of the median HU value, were overwritten.
- **Beam specific Water-equivalent-path length, ITV**_**WEPL**_: To assess the needed margin to overcome the range uncertainties caused by anatomy variations along the treatment, an ITV considering the changes of the WEPL over the set of weekly-CTs, ITV_WEPL,_ was determined. To this end, we employed the method defined by Graeff et al. [11], using the 4D extension of the treatment planning system (TPS) TRiP98 [12, 13], TRiP4D. In short, the HU changes along the specific beam direction in the set of weekly-CTs was converted into range changes, which led to the ITV_WEPL_. The latter was then optimized on the basis of these changes, to ensure adequate CTV coverage for the whole imaging set.

The definition of a Planning Target Volume (PTV) was out of the scope of this work as safety margins for set-up uncertainties will be subject of a separate investigation. Likewise intra-fractional motion uncertainties were also not considered.

### Treatment plan optimization and forward dose calculations

Plans were optimized using the research TPS TRiP98 [13] where the same beam base data and RBE input tables [12] were used as for our commercial system in clinical use, Syngo® RT Planning (Siemens Oncology Care Systems, Germany). The optimization technique used was intensity-modulated particle therapy (IMPT) with the ITV as the target to get full coverage of the CTV with a biological dose of 15 x 3 Gy (RBE). The biological dose calculation uses the local effect model [14]. The spinal cord, kidneys, stomach and bowel were considered organs-at-risk (OARs) and treatment plans were tailored to respect the tolerances suggested by the literature [15]. The α/β-ratio equal to 2 was used for all the various organs.

The IMPT optimization was performed with a reduced number of constraints in a way to preserve relatively high dose homogeneity in the single beams, preventing high gradients in the target volume, and avoiding the need of patch techniques. In the Additional file 1 a comparison between SFUD and IMPT plans for a set of patients is provided. This allows to demonstrate that IMPT plans with comparable homogeneity to SFUD plans and without compromising the plan quality can be achievable.

**Fig. 1:**
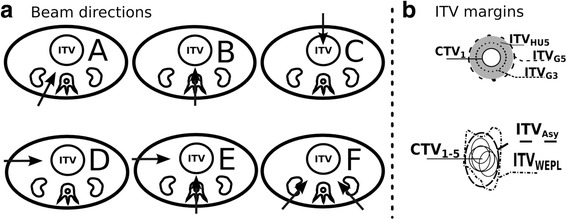
Plan parameters under evaluation. **a** Beam geometries with 1 field (*A-D*) and 2 fields (*E,F*). **b** Applied ITV-concepts in the optimization stage

For each patient six different beam geometries were optimized (Fig. 1) using pencil beams with 10 mm of FWHM, a raster spacing of 3 x 3 mm in lateral direction and an iso-energy slice spacing of 3 mm. Our raster scanning technique is characterized by a synchrotron spill structure of 5 s, a sweep time of 60 m/s and no rescanning is currently implemented.

Based on the optimized plan for the CT_plan_ the forward calculation was performed for the following weeks by application of the raster-scan sequence on the registered CT.

### Data analysis

The CTV coverage was determined by the volume that receives more than 95% of the prescribed dose (V95_CTV_). Additionally the CTV homogeneity dose (H_CTV_) was calculated by the difference of the dose given to 5% and 95% of its volume, H_CTV_ = D5 ‐ D95.

The concept of the ITV_WEPL_ was applied to evaluate the need of asymmetrically defined ITV to increase the plan robustness for patients under larger range changes. Using the TPS TRiP4D the ITV_WEPL_ was determined per field from the set of weekly-CTs and contours. The field-specific ITV_WEPL_ was evaluated by comparison with the CTV and ITV_G5_, through the volumetric changes and the ratio of the overlapping volumes (Dice Similarity Coefficient, DSC [16]). To extract the information of the margins direction and size we evaluated the variation of the center of mass (COM) and the Hausdorff distance (HD) between ITV_WEPL_ and CTV [16], which identifies the largest of all distances from one of the contours to the closest point on the other contour [17]. To reduce the patient-specific influence when applying this concept to other patients, the 95^th^ percentile of the HD was used as evaluation metric, HD_95_.

## Results

**Fig. 2:**
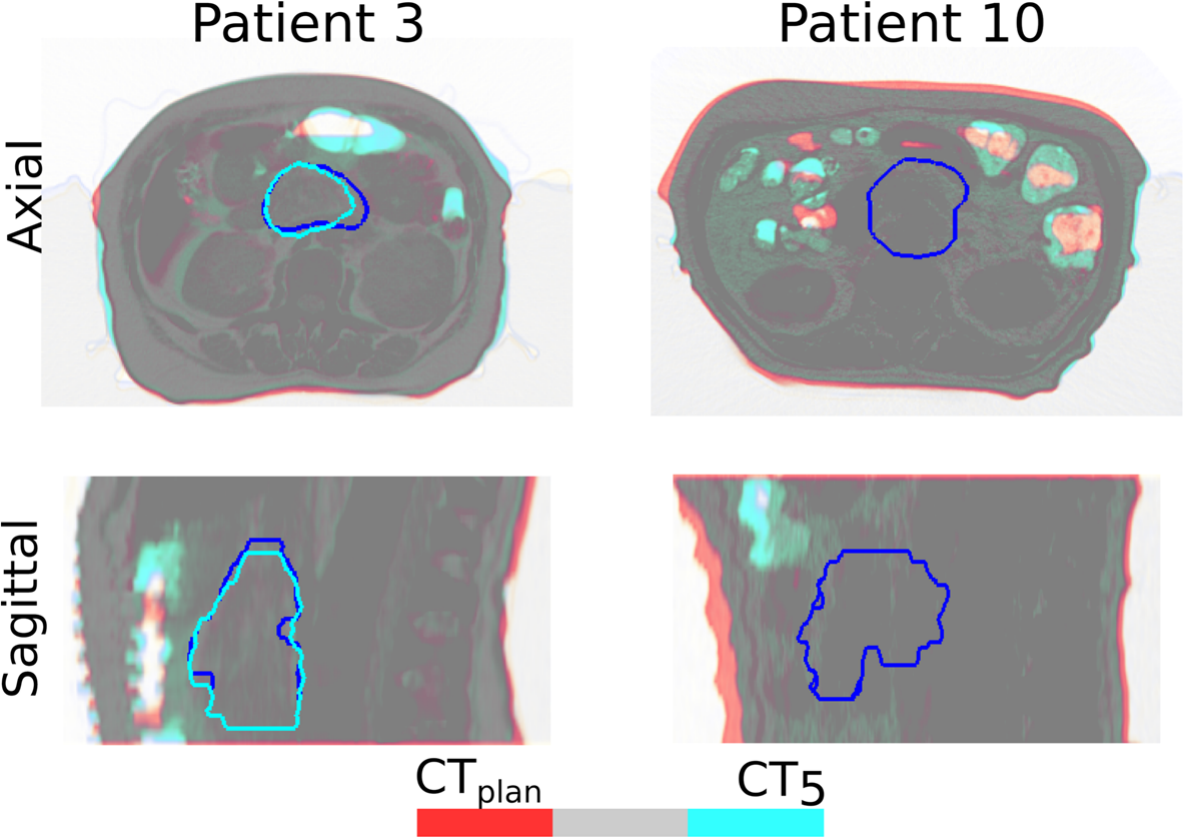
Overlay of the planning CT (*red*) and the CT of the week 5 (*blue*) for the patient 3 and 10. The grey color means perfect overlay between the patient anatomy and the *red/blue* color the region with anatomy changes (i.e. bowel, stomach and weight loss). The *dark blue* contour represents the planning CTV, while the *light blue* the CTV of the week 5 CT

The impact of anatomy and range changes on the target coverage and dose homogeneity among beam geometries margins concepts, patients, and fractions were analyzed. Furthermore, the CTV and COM changes along the treatment were analyzed and used to identify the reason for the dose degradation (e.g. CTV shrinkage, shift of CTV COM due to bowel volume, etc.). These data are available in the Additional file 1. Figure 2 shows two examples of the observed anatomical changes.

The plans with multiple beams showed an equally distributed and homogenous dose per beam i.e. each beam contributes to the total dose with (50.0 ± 2.5)% of the number of particles and a homogenous dose distribution per beam is observed, as consequence of a moderate IMPT optimization.

### Inter-fractional changes and Beam Geometry Robustness

**Fig. 3:**
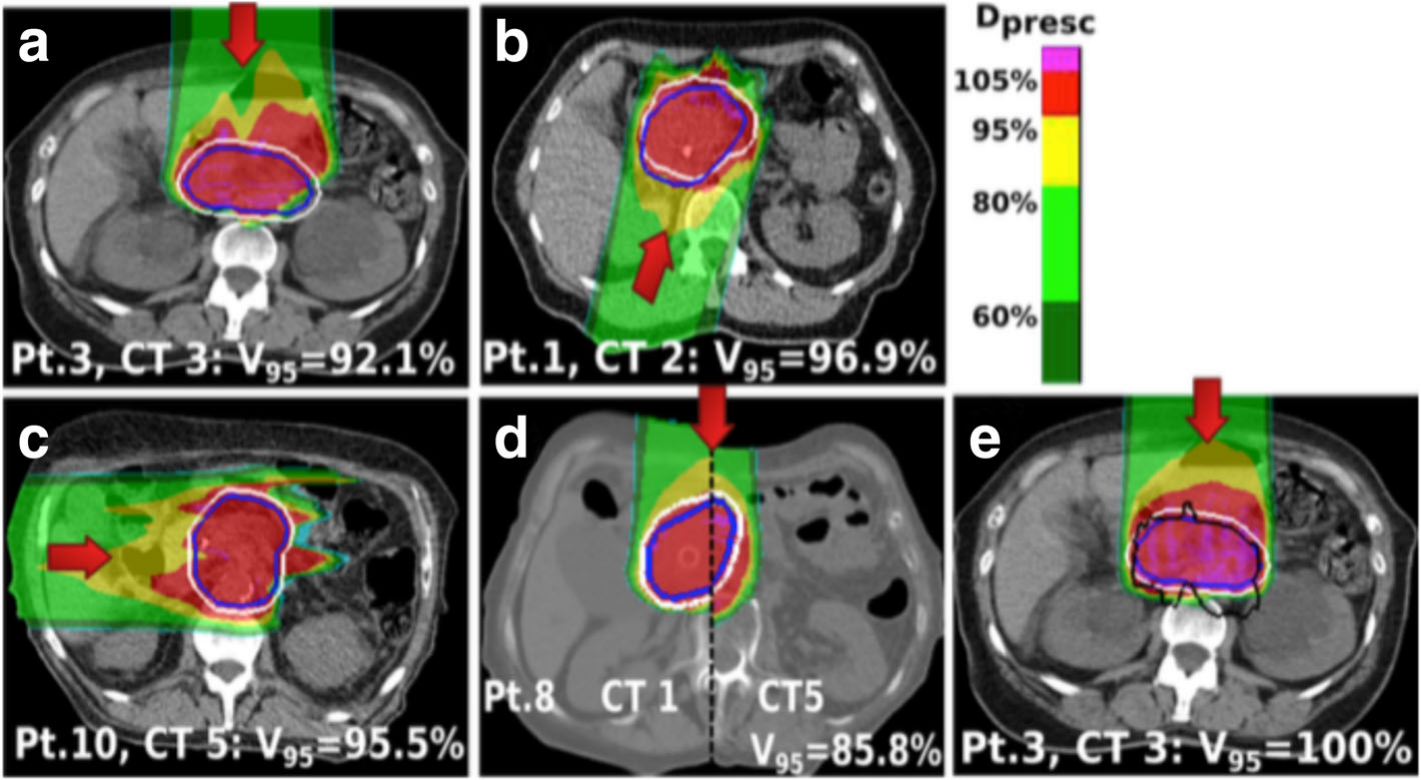
Forward dose distribution of the optimized plan for the ITV_G5_ (*white contour*) in one of the weekly CTs. Different inter-fractional changes are disclosed for analysis of the CTV (*blue*) V95_CTV_: (**a**) Patient 3, for which the use of an anterior beam results in CTV under-dose due to changes in the bowel/stomach, (**b**) Patient 6, the oblique posterior beam does not overcome the tumor shift and deformation. **c** Patient 10, irradiation with a lateral right beam, where the range changes resulted from the bowel and liver position led to health tissues overdose. **d** Patient 8, an anterior beam is not robust to the accentuated weight loss (visible the comparison of the distribution in the first and last week CT). **e** Dose distribution for the same CT and patient as in the figure a) but showing the retrospectively determined ITV_WEPL_ (*black contour*) in comparison to the ITV_G5_

The beam geometry showed a substantial impact on target coverage and homogeneity over the treatment course with the configuration C and D (Figs. 1 and 3) showing the worst average coverage as well as standard deviations along the treatment (Table 1). These values are related with the observed range uncertainties in the anterior abdomen region due to the inter-fractional variability of the anatomy (bowel, stomach, weight loss). Figure 3 shows the dose distribution impact in one of the follow-up CTs for four patients as representative of these inter-fractional changes contributors. V95_CTV_ for non-robust geometries decreased by up to 30% and 28%, for a 3 mm and 5 mm margin, respectively. This extreme effect was detected, as example, in a patient with larger weight loss (patient 8, Fig. 3d) and in a case with strong bowel variation (e.g. patient 3 and 10, Fig. 3a and c).

**Table 1:** V95_CTV_ mean and standard deviation over all patients and per beam incidences and ITV-concepts

V95 (mean ± st.deviation) of the CTV						
ITV Concept & Beam Configuration(A-F)	A	B	C	D	E	F
ITV_0_	95.6 ± 2.8	96.4 ± 2.7	88.7 ± 7.0	90.8 ± 5.6	98.0 ± 2.0	98.9 ± 1.5
ITV_3_	98.1 ± 1.7	98.6 ± 1.6	92.6 ± 6.4	94.0 ± 4.2	98.9 ± 1.7	99.5 ± 1.2
ITV_HU3_	98.1 ± 1.6	98.6 ± 1.6	92.6 ± 6.3	94.2 ± 4.1	99.1 ± 1.4	99.5 ± 1.2
ITV_5_	99.2 ± 1.2	99.4 ± 1.2	95.8 ± 5.2	96.7 ± 3.1	99.4 ± 1.3	99.6 ± 1.2
ITV_HU5_	99.2 ± 1.2	99.4 ± 1.2	95.8 ± 5.1	96.8 ± 3.0	99.4 ± 1.2	99.6 ± 1.2
ITVAsy	-	-	97.5 ± 3.9	98.1 ± 2.1	-	-

### Impact of margins

**Fig. 4:**
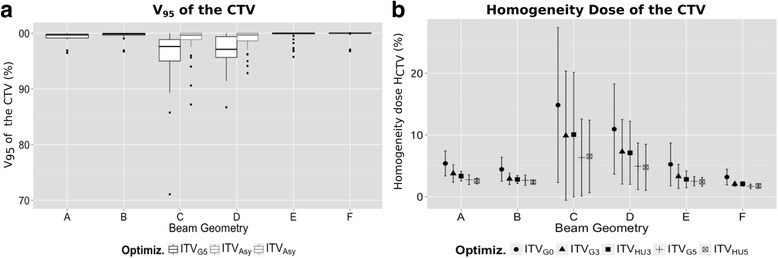
Evaluation of the CTV dose from the weekly dose distribution obtained from different optimizations. **a** V95_CTV_ of the plan optimized to the ITV_G5_ (dark grey boxes) and using the ITV_Asy_ for the non-robust geometries. Each box represents 25-75% of the data, with the median value represented as the solid line and the outlier as the dots. **b** Mean and standard deviation of the H_CTV_

The selection of robust beam geometries (as A B, E and F) together with the use of geometric margins showed to be able to mitigate some of the effects of the inter-fractional range changes, Table 1 and Fig. 4, where the use of the ITV_G5_ was enough to keep the mean V95_CTV_ above 99%. From the outlier of the Fig. 4a) is concluded that even for the more robust geometries there are cases of patients and isolated fractions with dose degradation. The analysis of these particular cases, was found to correspond to patients with changes in the tumor volume and COM (e.g. Fig. 3b). To overcome these situations the use of two oblique posterior beams showed to be more robust.

Other analyzed option to increase the plan robustness was the use of symmetric range-equivalent margins ITV_HU_. The results (Table 1 and Fig. 4a) showed that the used of 3 and 5 mm HU-uniform was not sufficient to mitigate the extreme range changes in the anterior and lateral beam direction, without significant improvement over the geometric concept.

### Asymmetric margin analysis

The obtained ITV_WEPL_ that includes the range variations along the weekly-CTs, represent how asymmetrically the ITV needs to be defined to maintain the CTV coverage. A representative example is shown in 3e.

The ITV size and shape necessary to keep the CTV coverage does not need to be volumetrically larger than a 5 mm expansion with the ITV_WEPL_ volume (−26.0 ± 6.3)% smaller than the ITV_G5_, as result of tight margins in the lateral direction to the beam and a margin increase for the non-robust beam geometries in the beam direction (depth).

The margin size and expansion direction were assessed from the COM variation and HD_95_ value. The mean V95_CTV_ over the course of the treatment when no margins are applied was used as metric of the inter-fractional changes per beam direction. Its correlation with the variation of the DSC and the HD_95_ between the CTV and the ITV_WEPL_ led to the selection of the HD_95_ as indicator of range changes due to a positive linear relation (*r* = 0.78) versus the smaller correlation coefficient of the DSC (*r* = 0.63).

**Fig. 5:**
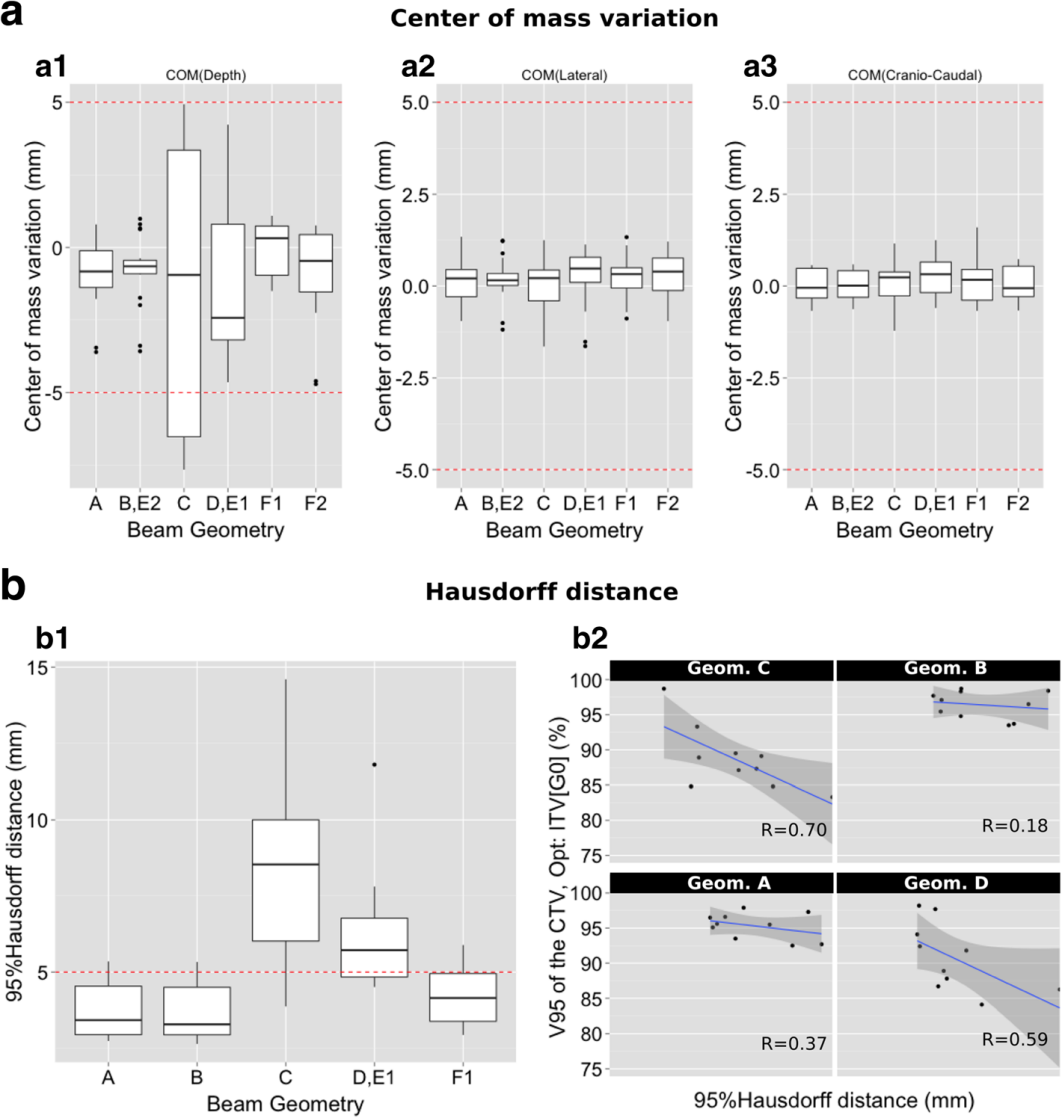
(*a1*-*a3*) Variation of the ITV_WEPL_ COM relative to the CTV in the longitudinal (*a1*), lateral (*a2*) and superior-inferior (*a3*) direction with respect to beam’s-eye-view. *b1*) HD_95%_ as function of the beam geometry, with each box showing 25-75% of the data. *b2*) Correlation, per beam direction, between the CTV mean dose for the weekly CTs (plan optimized to the ITV_G0_) and the HD_95_

The analysis of the obtained ITV_WEPL_ is presented in terms of median and quartiles values in the Table 2 and Fig. 5. For non-robust geometries (C and D) the margin to apply to the CTV to cover the changes can be up to 10 mm in depth larger in the proximal edge of the beam for the most patients, while the lateral direction to the BEV requires less than 5 mm. Based on these results, new plan optimizations were performed for an asymmetric ITV (ITV_Asy_) that covers 75% of the dose distributions, corresponding to a margin in the proximal direction of 10 mm and 7 mm, and of 6 mm and 3 mm in the distal direction to cover the COM variations for the geometry C and D, respectively. The delivery of these new plans to each weekly-CT, showed an improvement of the V95_CTV_, Fig. 4a.

**Table 2:** Evaluation of the calculated ITV_WEPL_ by: volume comparison with the ITV_G5_ (mean, minimum and maximum are shown); directional expansion relative to the CTV (HD_95%_); COM variation relative to the CTV. The reported values as Q_25%_ and Q_75%_ correspond respectively to the first and third quartiles of the data

	ITV_WEPL_ Characterization				
Beam Configuration (A-F)	ΔV ITV_5_-ITV_WEPL_		CTV expansion (mm)	ΔCOM. Depth (mm)	ΔCOM. Lateral (mm)
	Mean (%)	[Min-Max] (%)	(Q_75%_ of HD_95%_)	[Q_25%_;Q_75%_]	[Q_25%_;Q_75%_]
A, F1	−28.0	[−35.3,-22.6]	4.5	[−1.2; −0.3]	[−0.2;0.3]
B,E2	−27.9	[−36.5,-22.1]	4.5	[−0.8 -0.5]	[0;0.2]
C	−16.9	[−30.5,-8.4]	10	[−5.3;2.8]	[−0.2;0.5]
D,E1	−26.9	[−37.2,-15.5]	6.8	[−3.1;0.4]	[0.1;0.7]
F2	−28.7	[−35.9,-21.4]	5.0	[−0.7;0.7]	[−0.1;0.5]

For robust geometries the analysis showed to be suitable the use of a symmetric margin where 5 mm is enough for 75% of the fractions.

## Discussion

For the analyzed patients the tumor inter-fractional changes are in agreement to the findings by Liu et al. [18] who report changes in the COM in the order of 1–2 mm with substantial changes in the tumor volume. Anatomical changes in the healthy tissues were also detected along the course of the treatment, with density changes in the bowel and stomach and weight loss, resulting in drastic reductions of the V95_CTV_ and homogeneity. Kumagai et al. [10] pointed out variations on the D95 _CTV_ of 10% due to intra-fractional bowel changes using a passive delivery system, which matches with our results using a single beam in the anterior or lateral direction in the presence of inter-fractional changes. An interesting study of Houweling et al. [19] used alternatively the routinely acquired cone-beam CT (CBCT) to assess the inter-fractional changes in the patient anatomy, through dose calculations in a CT, obtained by deformable registration of the planning CT to these CBCTs. This method results in an increase of the available data but the deformable registration adds new uncertainties to the evaluation.

The influence of the range changes on dose to the OARs was not part of our analysis and further investigation need to be included especially for geometries where the OARs are in the beam distal edge.

We have demonstrated that the impact of inter-fractional changes can be reduced to a large extent with an adequate selection of the beam geometry and ITV-margins. For the analyzed treatment fractions and patients the use of a symmetric margin of 5 mm and two posterior oblique beams was enough to keep the CTV coverage above 95%, with 10% of the cases with a V95_CTV_ of 95-98%. Nevertheless, cases with substantial changes in the CTV shape or location were detected, leading to a decrease in dose coverage (outliers in Fig. 4a). In these cases, treatment plan adaption or re-planning might be an option. Moreover, our results only considered uncertainties resulting from anatomy changes and the uncertainties from the patient positioning were neglected. Regarding the patient positioning, studies from Jayachandran et al. [20] showed that bone-matching registration might not accurately predict the tumor location, and therefore suggests the use of fiducial markers combined with daily soft-tissue imaging.

The definition of PTV margins is beyond the scope of this work which aimed to isolate the inter-fractional motion impact. With exception of errors from the rigid registration, other systematic errors, as example uncertainties in Hounsfield units or patient positioning, do not have an impact on our simulation results. However, they need to be taken into account for clinical implementation.

An important scope of the study was to suggest clinically feasible approaches to increase the plan robustness to account for range uncertainties. From the first attempt with a symmetric range-uniform concept, the results showed that a small margin of 3 or 5 mm was not enough to overcome the uncertainties from non-robust beam configurations. Hence, the use of asymmetric field specific margins was evaluated, which was already investigated by Park et al. [21] as an approach to mitigate range uncertainties. Also, Miki et al. [22] investigated the use of field-specific target and OARS but for the case of intra-fractional changes. In our study, the obtained ITV_WEPL_, based on Graeff et al. [11] study, preserves the CTV coverage over the set of weekly-CTs, including the inter-fractional anatomical changes. The obtained ITV_WEPL_ matched with the literature that suggests small lateral margins and larger margins in the direction of the low density tissues, that here correspond usually to the proximal direction [21, 23].

Note that the ITV_WEPL_ concept cannot be directly used in the clinical routine since the complete information of the inter-fractional changes along the treatment course is not readily available at the planning stage. However, we have used this approach to guide the definition of an asymmetric standard margin for future patients. The suggested asymmetric margins were tested for the non-robust geometries and showed a V95_CTV_ improvement face to the symmetric concept. The validation of this concept in a different cohort of patient remains to be shown and will be investigated in a future study. Furthermore, this concept could be applied clinically e.g. in a boost treatment, where several planning CTs have been obtained in the first treatment sessions with photons.

Our study was tailored to improve the treatment plan robustness against inter-fractional changes using IMPT optimized plans. However, a comparison of the plan quality using SFUD and IMPT is suggested prior to the implementation of this technique in others facilities, since the degree of plan modulation can compromise the robustness. In our study, all the plans represented reduced modulation. Therefore the results might be extended to SFUD optimizations.

Intra-fractional changes were not investigated and will require an additional expansion of the target volume [24]. Larger margins alone are not a solution for scanned ion therapy since interplay effects will have an impact on the dose coverage and homogeneity throughout the complete volume [25, 26]. Shiomi et at. [27] evaluated, how the use of specific beam directions might increase the plan robustness for the case of passive and active delivery systems. Moreover, a complementary approach is the use of abdominal compression to reduce the motion amplitude and additional beam rescanning to mitigate the interplay effect [28]. There is no consensus on the quantification of intra-fractional motion in pancreas [18, 29] among the studies, and a separated study will be conducted to assess the need of mitigation techniques. This might include the use of beam-gating [30] or other modified beam delivery strategies, as well the use of 4D optimized plans [31] or quantification of range variations based on daily CT imaging [32].

## Conclusion

In conclusion, the combination of two oblique posterior beams for scanned carbon-ion treatment of pancreatic cancer with the simple concept of an isotropic CTV-ITV margin of 5 mm can substantially reduce the dosimetric impact of inter-fractional changes and yields to acceptable dose coverage for most patients and fractions. The use of WEPL-based margins can reduce the required margins even more, but this technique will require a daily monitoring of the anatomical changes. Therefore, range variations may be large and routine soft-tissue imaging and adaption strategies will likely improve the treatment. Population-based asymmetric ITV margins may be a feasible clinical strategy to account for density changes and to reduce the irradiated volume as well as to assess the validity of the selected margins for a defined beam path direction.

## Additional file

Additional file 1: Supplementary Figure and Tables. (DOC 831 kb)

## Abbreviations

COM: Center of mass
CT: Computed tomography
CTV: Clinical target volume
DSC: Dice similarity coefficient
GTV: Gross tumor volume
H_CTV_: Homogeneity dose
HD: Hausdorff distance
HU: Hounsfield unit
IMPT: Intensity-modulated particle therapy
ITV: Internal target volume
PTV: Planning target volume
r: Person correlation index
RBE: Relative biological effectiveness
TPS: Treatment planning system
WEPL: Water-equivalent range path
