# Supplementary material for: Planning strategies for inter-fractional robustness in pancreatic patients treated with scanned carbon therapy
Source: Radiat Oncol. 2017 Jun 8;12:94. doi: 10.1186/s13014-017-0832-x (PMC5465513; doi:10.1186/s13014-017-0832-x)
Supplement: Additional file 1: — Supplementary Figure and Tables. (DOC 831 kb) [file 13014_2017_832_MOESM1_ESM.doc]

# Additional file

Additional file 1: **Table S.1:** Patient information of the initial CTV volume, and its variation in volume (ΔV) and position (Δ vector displacement) over the treatment course.

| **Patient** | **CTV Initial Volume (V_plan_) (cc)** | **Δ V (%)** | **Δ vector displacement (mm)** |
| --- | --- | --- | --- |
| **1** | 45992 | -10.4 ± 6.5 | 3.7 ± 2.1 |
| **2** | 32317 | -8.2 ± 8.2 | 2.3 ± 0.2 |
| **3** | 62952 | -1.0 ± 2.5 | 2.2 ± 1.8 |
| **4** | 26348 | -4.9 ± 2.7 | 2.5 ± 0.9 |
| **5** | 44485 | 0.2 ± 0.8 | 1.2 ± 0.8 |
| **6** | 39225 | 2.3 ± 0.5 | 2.2 ± 0.4 |
| **7** | 37129 | 1.8 ± 4.1 | 1.4 ± 0.1 |
| **8** | 43489 | 2.0 ± 2.1 | 0.5 ± 0.2 |
| **9** | 33120 | -6.2 ± 0.8 | 1.8 ± 0.1 |
| **10** | 80388 | -1.1 ± 0.7 | 0.3 ± 0.2 |
| **Mean ± Std.dev.** | **-** | **-2.6 ± 5.5** | **1.8 ± 1.3** |

**S.2.** **Additional analysis:** Comparison of the plan modulation of optimized plans using SFUD and IMPT

The robustness of a treatment plan to uncertainties (inter- and intra-fractional motion, patient positioning…) is strongly affected by the plan modulation, i.e., how homogeneous is the dose distribution between neighboring voxels. Two metrics to assess the modulation of the plan were used, the normalized standard deviation of the number of particles per slice ($\overline{\sigma{}_{IES}}$) and the modulation index (MI). The $\overline{\sigma{}_{IES}}$ is described by the equation (a), where *mean_ies_* is the mean number of particles in a specific iso-energy slice (IES) and σ_IES_ is the respective standard deviation in a total of *n* iso-energy slices. The MI calculation was based on the concept developed by Webb [1] for IMRT, and is given by equations (b1-2). It is calculated considering the magnitude of the difference between the intensity from neighboring raster points (rp), ∆ = |I_rp_ −I_rp−1_| and the number of raster points (np) that in each IES are above a threshold of the standard deviation of the IES (defined as δ = 1.2), N. Hence, the area of this spectrum of deviations, i.e. area below the F function, gives the degree of modulation, MI.


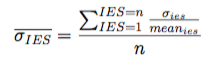
(a)


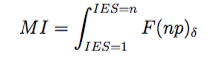
(b1)


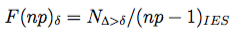
(b2)

These parameters were evaluated in the context of another study and a subset of four patients either optimized with SFUD or IMPT are here presented to justify that IMPT might result in plans with comparable modulation to SFUD plans. Table S.2.1 and figure S.2.2 show the results obtained for these four patients.

It was observed that these parameters trend to be higher for IMPT plan were tight constraints were used during optimization, reducing their robustness to range changes. Therefore, the influence of the plan modulation was considered, since the entire dataset was optimized keeping a low plan modulation, achievable with a rational number of constraints.

Additional file 1: **Table S.2.1.:** Evaluation of the dose distribution for four patients in terms of CTV dose homogeneity (D_5_-D_95_ = difference between the dose covering 5% and 95% of the CTV volume) and the plan homogeneity in terms of normalized standard deviation of the number of particles ($\overline{\sigma{}_{IES}}$) and modulation index (MI).

| **Patient** | **N° & hardness of the**  **Constraints** | **SFUD** | **IMPT** | **CTV dose**  **Homogeneity (%)** | $\overline{\sigma{}_{IES}}$ | **MI** |
| --- | --- | --- | --- | --- | --- | --- |
| **H2** | low | x |  | 12.65 | 3.6 | 9.9 |
| **H8** | low |  | x | 14.77 | 3.9 | 4.1 |
| **H14** | low |  | x | 10.20 | 4.3 | 3.0 |
| **H7** | high |  | x | 7.82 | 5.3 | 17.8 |

Additional file 1: **Figure S.2.2.:** Patient H2 (left) was optimized using SFUD while patient H8 (right) with IMPT. Both plans showed comparable CTV dose homogeneity and standard deviation of the number of particles.


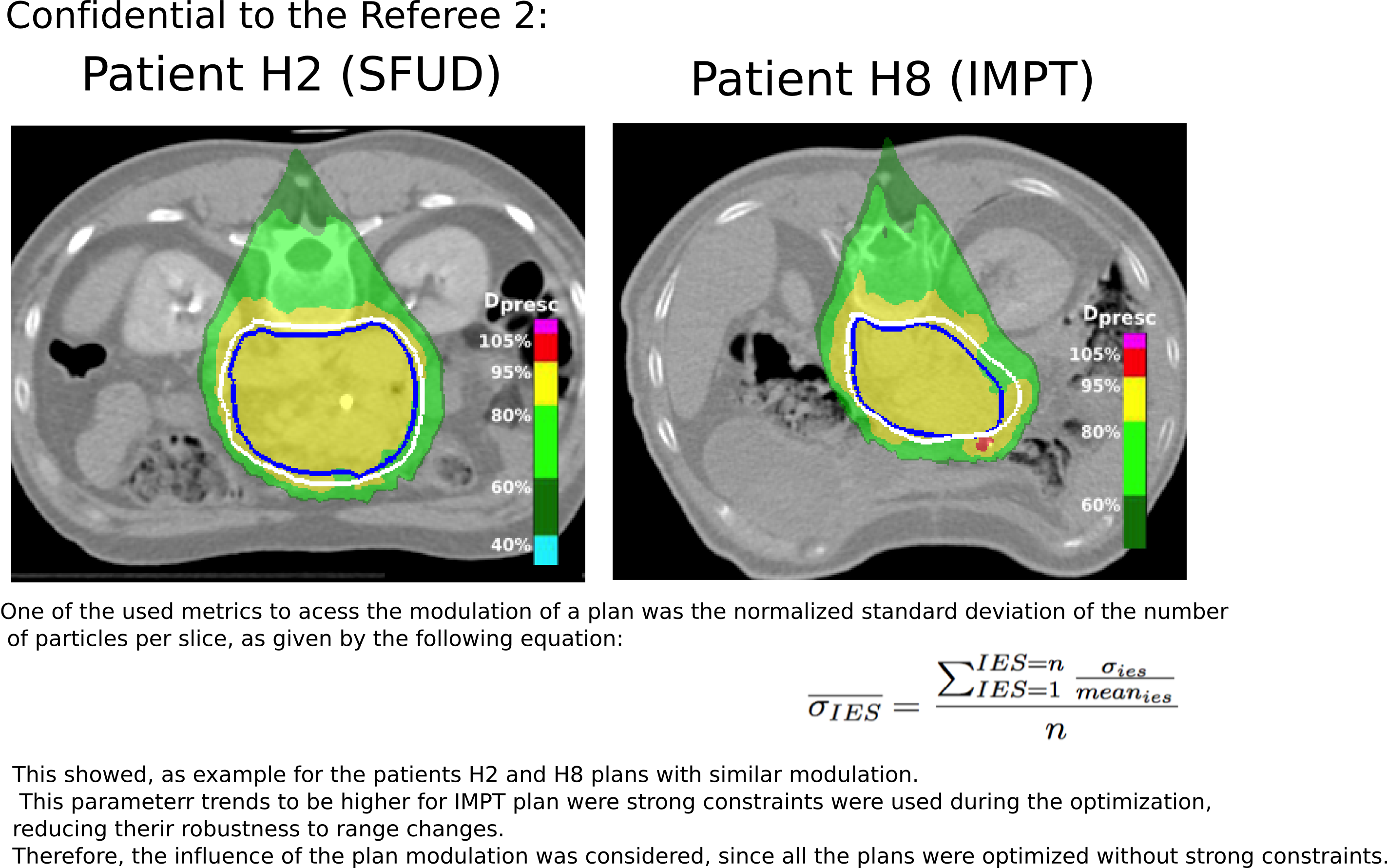


----

[1]. Webb, S. Use of a quantitative index of beam modulation to characterize dose conformality: illustration by a comparison of full beamlet IMRT, few-segment IMRT (fsIMRT) and conformal unmodulated radiotherapy. Physics in medicine and biology 48.14, p. 2051, 2003
